# Forgiveness, Marital Quality, and Marital Stability in the Early Years of Chinese Marriage: An Actor–Partner Interdependence Mediation Model

**DOI:** 10.3389/fpsyg.2018.01520

**Published:** 2018-09-04

**Authors:** Qiong He, Mengyu Zhong, Wei Tong, Jing Lan, Xiaomin Li, Xiaoyan Ju, Xiaoyi Fang

**Affiliations:** ^1^Institute of Developmental Psychology, Beijing Normal University, Beijing, China; ^2^Department of Social Work, China Youth University of Political Studies, Beijing, China

**Keywords:** forgiveness, marital quality, marital stability, Chinese couple, APIMeM

## Abstract

Based on the Vulnerability Stress Adaptation model, this study examined the relationship between forgiveness and marital stability, and provides a first look at the mediating role of marital quality in this association during the first 3 years of marriage based on three annual waves of data collected from 268 Chinese couples. Tests of actor–partner interdependence mediation models revealed direct effects of decisional forgiveness and emotional forgiveness on the concurrent levels of marital stability for husbands, and indirect effects of emotional forgiveness on the concurrent and longitudinal levels of marital stability through marital quality for both husbands and wives. There was also an indirect effect of wives’ emotional forgiveness on concurrent and longitudinal levels of husbands’ marital stability through their wives’ marital quality. Thus, emotional forgiveness, rather than decisional forgiveness, contributes to longitudinal levels of marital stability through marital quality. Theoretical implications and future directions for research are discussed.

## Introduction

Marital stability, which refers to affective and cognitive states along the related actions that are precedent to terminating a relationship (Booth et al., 1983; Amato et al., 2007), is a key indicator of well-being (Karney and Bradbury, 1995). A number of studies have shown that separated and divorced couples experience greater risk for mental and physical health problems (Amato, 2010; Wang et al., 2015), as well as can have strong negative consequences for their children, such as impacting their children’s mental health, academic and social performance (Schramm, 2006; Amato, 2007; Lansford, 2009). Since marriage instability is detrimental to the family unit and society (Orathinkal and Vansteenwegen, 2006), the importance of identifying the factors that promote marital stability is overwhelmingly obvious.

Although there is substantial literature on the interpersonal predictors for divorce and relationship stability (Gottman and Notarius, 2002; Amato, 2010), several important gaps can be identified in the field. First, previous research has focused extensively on the impact of negative factors, and has not paid sufficient attention to the role of positive interpersonal processes in marriage dissolution, such as forgiveness, commitment, and sacrifice (Reis and Gable, 2003; Fincham et al., 2007). Previous studies have revealed that forgiveness is the cornerstone of a successful marriage (e.g., Fincham et al., 2006; Worthington, 2009) as it builds a strong connection to overcome negative outcomes by cultivating positive affect and behavior (Worthington et al., 2007; David and Stafford, 2015). Second, while researchers studying continuously healthy relationships have defined full forgiveness as both reducing unforgiveness (e.g., a cognitive decision to forgive) and increasing positive emotion (e.g., emotional forgiveness) (Worthington, 2005), emotional forgiveness has not received as much attention. Third, forgiveness theories have rarely been connected to broader theories in couple research (Braithwaite et al., 2016), such as the Vulnerability Stress Adaptation (VSA) model. According to the VSA model, the adaptive process has effects on marital stability via marital quality (Karney and Bradbury, 1995), and evidence that forgiveness as an adaptive process to resolve the challenges of marriage has been linked to marital quality, which are needed to retain marital stability (Hall and Fincham, 2006; Fincham et al., 2007; Entezar et al., 2011; Braithwaite et al., 2016; Kato, 2016), but the associations among forgiveness, marital quality, and marital stability remains relatively unexplored. Fourth, whereas interdependence theory highlights the importance of considering both actor effects and partner effects, few studies have taken a dyadic approach when examining forgiveness (Fincham, 2009). Fifth, research in this field has been conducted extensively with samples of Western couples from individualistic cultures. While collectivistic cultures emphasize forgiveness as an important step in restoring a relationship “toward harmony” (Exline and Baumeister, 2000, p. 138), the association between forgiveness and marital stability has rarely been examined with samples from these cultures.

The current study sought to contextualize the associations between forgiveness, marital quality, and marital stability within the broader theoretical framework of the VSA Model in the early years of Chinese marriage, by employing a longitudinal design and dyadic approach (Actor–Partner Interdependence Mediation Model [APIMeM]; Ledermann et al., 2011). The study had two goals: (a) to examine the effects of one’s own and one’ partners’ forgiveness on the concurrent and the longitudinal levels of one’s own and one’s partners’ marital stability, (b) to identify whether one’s own and one’s partners’ marital quality is a potential mediating mechanism in such associations.

### Forgiveness and Marital Stability

Since Fenell (1993) found that forgiveness is integral to a long-lasting marriage, forgiveness has received increasing attention from researchers because of its positive influence on marital quality and marital stability via the adaptive process by mending inevitable injuries and transgressions (e.g., Fenell, 1993; McCullough et al., 1998; McNulty, 2008; David and Stafford, 2015). Despite there is a lack of consensus on the definition of forgiveness, researchers agree that forgiveness involves a prosocial changing process toward the transgressor (McCullough et al., 2001; Li and Lu, 2017), in which negative motivation (e.g., unforgiveness) decrease and positive emotions increase (McCullough et al., 2003; Fincham et al., 2005). While several scholars proposed that the forgiveness process incorporates an intellectual decision to forgive, as well as an emotional forgiveness component (Enright, 1996; Strelan and Covic, 2006), empirical evidence in respect to the different roles between decisional and emotional forgiveness on marital stability is rare. Therefore, this study focused on forgiveness as two distinct, but related processes, in continuing relationships: decisional forgiveness, which is a decision to change one’s behavioral intentions in order to eliminate revenge and to restore interaction; and emotional forgiveness, which involves changes in emotion, motivation, cognition, and eventually behavior (Worthington, 2006). For example, an individual may be angry when offended by his or her partner. The individual may make a decision to forgive the offender, meaning that he or she intends to act toward the partner in ways that are more positive (e.g., will be friendly toward the partner). However, even though the individual has made a decision to forgive, they may not experience emotional forgiveness (e.g., resentful and angry filled) (Hook et al., 2009). While emotional forgiveness meaning that toward the partner, his or her would be replacement of negative emotions (e.g., less anger or less hurt) by cultivating positive emotion (e.g., more compassion or less love). Moreover, emotional forgiveness involved in the cognitive aspects of forgiveness that leads to truly forgive the partner by forgetting of offense relevant traits (Lichtenfeld et al., 2015).

A number of studies have shown that decisional forgiveness (e.g., reducing unforgiveness), is associated with higher levels of relationship stability (e.g., McCullough et al., 1997; Wieselquist, 2009; Fehr et al., 2010), but very few studies, to date, have explicitly investigated the effects of emotional forgiveness on a couples’ marital stability. There has only been some indirect evidence of the effects of decisional and emotional forgiveness on marital quality. For instance, Chi (2011) examined forgiveness by assessing decisional forgiveness and emotional forgiveness, and results showed that both processes of forgiveness substantially and prospectively correlated with marital quality among Chinese couples. Therefore, the roles played by the two distinct processes of forgiveness in longitudinal relationship stability remains to be identified.

Moreover, interdependence theory indicates that couples are interdependent on outcomes of their behavior (Kelley and Thibaut, 1978). Whereas it is worth noting that one’s attributes and behaviors can affect one’s partner’s outcomes (Rusbult and Van Lange, 2003), research examining the impact of forgiveness on marital quality or marital stability has been limited to the individual level of analysis. Some cross-sectional studies have documented the association between wives’ forgiveness and both their own and their partners’ marital outcomes. For instance, Karremans et al. (2003) explored the relationship between forgiveness and marital quality in couples and found that while wives’ propensity to forgive predicted both their own and their partners’ future marital quality, this was not the case for husbands. In line with interdependence theory, this study employs a longitudinal design and takes a dyadic approach by obtaining information from both husbands and wives. This tactic affords for the identification of how a partner’s forgiveness works independently, and in combination with one’s own forgiveness to both impact marital stability as well as to analyze partner effects.

### Potential Mediating Mechanism: Marital Quality

Although previous studies have documented that forgiveness is linked to relationship stability (e.g., Fincham, 2009; Wieselquist, 2009), no previous evidence exists about a longitudinal relationship between forgiveness and relationship stability over the course of long-lasting, committed relationships, such as marriage, and the mediating mechanisms underlying this association is unclear. However, a promising potential mediating variable that has not been investigated is marital quality. Marital quality has been theorized to be based on pathways through which forgiveness is related to marital stability. That is, as the VSA Model implies, adaptive processes are generally characterized by couples’ ability to adapt effectively to the challenges of marriage and influence marital stability via marital satisfaction (Karney and Bradbury, 1995; Hardy et al., 2015). Furthermore, Braithwaite et al. (2016) proposed that forgiveness would be considered an adaptive process in the VSA Model and empirical evidence supports these hypotheses. For example, numerous studies have indicated forgiveness as an adaptive process is associated with marital quality (Entezar et al., 2011; Safarzadeh et al., 2011) and marital stability (Hall and Fincham, 2006; Wieselquist, 2009). There is also evidence that marital quality is associated with marital stability (Karney and Bradbury, 1995; Le and Agnew, 2003). However, whether forgiveness contributes to marital stability by promoting marital quality in a longitudinal context has yet to be determined.

### Forgiveness and Marital Stability Among Chinese Couples

Marriage stability has played a critical role in Chinese society. Historically, marriage in China was simply not regarded as a means of enhancing the happiness of married couples (Liao and Heaton, 1992). Instead, it was a means of promoting the goals of familyism and group harmony. Chinese couples may have observed great barriers to leave a marriage due to the importance of loyalty and forgiveness in traditional beliefs (Tang, 2011). On the one hand, although less stigma is attached to divorce today than in the past, many people still value long-term marriage (Wong et al., 2014). Therefore, collectivists are characterized by making a decision to forgive in order to achieve reconciliation rather than emotional forgiveness of the offense (e.g., DiBlasio, 2000; Ripley and Worthington, 2002). On the other hand, in China, the crude divorce rate (per 1,000 population) increased significantly from 0.18‰ in 1978 to 2.8‰ in 2015 (Chinese Ministry of Civil Affairs, 2016). One key reason for this occurrence may be that the majority of young Chinese people getting married were born after the implementation of the “1979 One-Child Policy.” Their maturing experiences are often characterized by parental indulgence, which may contribute to their emphasis on expression of emotion and self-interest within interpersonal relationships (Wang and Fong, 2009). Therefore, in the face of partner transgressions, young Chinese couples may be more focused on emotional forgiveness in long-term marriage.

### Current Study and Hypotheses

On the basis of the above research on forgiveness, relationship quality, and romantic relationship maintenance, this study viewed forgiveness as an adaptive process that can be integrated into broader theoretical research, such as the VSA Model (Karney and Bradbury, 1995), and aims to shed light on the ways in which forgiveness affects marital stability through marital quality based on three annual waves of data obtained from Chinese couples. Consequently, this study had two main goals. The first goal of the study was to determine whether decisional forgiveness and emotional forgiveness play independent roles in concurrent and longitudinal levels of marital stability. Based on past research (McCullough et al., 1998; Fehr et al., 2010), we hypothesized that one’s own and one’s partner’s decisional forgiveness and emotional forgiveness, as two types of adaptive processes, will be positively associated with one’s own concurrent levels of marital stability.

Furthermore, in line with the VSA model (Karney and Bradbury, 1995), the second goal of the study was to address the aforementioned limitations in previous research by testing an APIMeM (Ledermann et al., 2011), in which spouses’ decisional forgiveness and emotional forgiveness was linked to their own and partners’ marital stability through their own and partners’ marital quality. Given that forgiveness in its actual sense emphasizes the elimination of a grudge by replacing the negative emotion with positive emotion in order to eventually feel at peace with their partner (Richards, 2002; Worthington, 2005), we hypothesized that spouses’ emotional forgiveness leads to the longitudinal levels of marital stability through marital quality.

## Materials and Methods

### Participants and Procedure

Data for this study came from the Chinese Newlyweds Longitudinal Study (CNLS) conducted between 2012 and 2014, a 3-year longitudinal study examining factors affecting the marital quality and stability of couples in China. The present study’s research procedures were approved by the Institute Review Board at the study’s home institution. Based on the family life cycle, newlywed has been considered as couples who were in the first 4 years of first marriage and marked by starting in marriage of the couple and ends at the birth of the first child (Anderson et al., 1983; McDaniel et al., 1990; Reis and Sprecher, 2009). Therefore, at Time 1, sampling was undertaken to identify couples who were within 3 years of their first marriage, without children, and living together in Beijing, China. Ultimately, 268 couples participated in this study, in response to posted announcements on websites and in communities. In the announcements, participants were informed that the study consisted of three repeated assessments at 12-month intervals.

At the time of initial data collection, couples had been married for an average of 13.59 (*SD* = 9.69) months. Husbands averaged 29.59 (*SD* = 3.25) years of age, reported a median monthly income of 7,000 RMB (*SD* = 6180.22; around US$1,049.07), and had 15.3 (*SD* = 2.2) years of formal education. Wives averaged 28.08 (*SD* = 2.51) years of age, reported a median monthly income of 5,000 RMB (*SD* = 3,996.03, around US$749.34), and had 15.5 (*SD* = 1.6) years of formal education. Most participants had at least some college education (husbands = 91.3%; wives = 95.4%), and participants had higher levels of income and education than the broader population (Beijing Bureau of Statistics, 2011).

Two years later, 209 of the 268 couples participated in assessment at Time 3, which resulted in a 78.0% retention rate. Of those couples, data from six couples were deleted because only one spouse in the couple participated in the follow-up study or because both spouses were missing more than one-third of values on the central measures. Thus, the final sample at T3 comprised 203 couples. To test for attrition effects, independent sample (attrited vs. retained) *t*-tests were conducted on all T1 variables of interest. There were two significant differences between attrited husbands and retained husbands in marital quality (*M*_attrited_ = 6.42, *SD* = 1.13, *M*_retained_ = 6.83, *SD* = 0.88, *t* = -2.73, *p* < 0.01, Cohen’s *d* = 0.40), and marital stability (*M*_attrited_ = 3.71, *SD* = 0.41, *M*_retained_ = 3.83, *SD* = 0.31, *t* = -2.62, *p* < 0.01, Cohen’s *d* = 0.33) at T1. On the basis of Cohen’s (1988) criteria, the magnitude of these Cohen’s *d* values was between “small” and “medium.” There was non-significant differences between attrited wives and retained wives on all T1 variables of interest.

At T1, T2, and T3, data were collected using the same assessments. Both husbands and wives were invited to the research lab to participate in the study. For couples who could not come to the lab, research assistants collected the data during a home visit (*n =* 44 couples, 16.4% of all couples). First, the study was described in general terms by a trained research assistant, and signed written informed consent was obtained from each participating couple. Then, husbands and wives separately completed self-report measures. Each of the couples was paid 100 RMB (approximately US$16) and given a small gift (e.g., a cup) at each time point for their participation in the study.

### Measurement

All measures used in the current study were originally developed for American couples. A team of graduate students majoring in human development and family studies who were fluent in both Chinese and English first translated these measures into Mandarin, and then another team of bilingual graduate students translated them back into English. The investigators also worked with the translators to revise items as needed until it was evident that the Chinese items had meanings equivalent to those of the English version.

#### Marital Stability

The five-item unidimensional Marital Instability Index (Booth et al., 1983) was used to assess marital stability. It is a behaviorally oriented assessment of the propensity to separate or get divorced (Yeh et al., 2006). For each item, spouses were to indicate their agreement with a statement (e.g., “Has the thought of getting a divorce or separation crossed your mind?” and “Did you talk about consulting an attorney about a possible separation or divorce”) on a 4-point scale ranging from 1 (never) to 4 (frequently). The responses on the Marital Instability Index were reversed and averaged to calculate a score ranging from 1 to 4, with higher scores reflecting greater relationship stability. Mean scores were calculated and used in analyses. Cronbach’s *α*’s in the current study were ranged from 0.73 – 0.76 and 0.81 – 0.85 for husbands and wives at all time point respectively.

#### Marital Quality

The six-item unidimensional Quality Marriage Index (Norton, 1983) was used to assess marital quality. For the first five items, spouses were asked to indicate their agreement with statements (e.g., “I really feel like part of a team with my partner”) on a 7-point scale ranging from 1 (very strong disagreement) to 7 (very strong agreement). The last item asked spouses to indicate how happy they are in their marriage, all things considered, on a 10-point scale from 1 (very unhappy) to 10 (perfectly happy). All six items were worded in a positive direction. Scores on the Quality Marriage Index ranged from 6 to 45, with higher scores reflecting higher relationship quality. Mean scores were calculated and used in analyses.

Cronbach’s *α*’s in the current study were ranged from 0.91 – 0.93 and 0.90 – 0.95 for husbands and wives at T1 and T2 respectively.

#### Decisional Forgiveness

The 8-item self-report Decisional Forgiveness Scale (DFS; Worthington et al., 2007) was used to measure the degree to which one had made a decision to forgive someone of a specific offense. Participants indicated their agreement with each item on a 5-point scale, from 1 (strongly disagree) to 5 (strongly agree). The DFS has two 4-item subscales, one indicating prosocial intentions toward an offender (e.g., “If I see him or her, I will act friendly”), and one indicating the inhibition of harmful intentions toward an offender (e.g., “I will try to get back at him or her” [reverse scored to indicate forgiveness]). Mean scores were calculated and used in analyses. Higher scores indicated higher levels of decisional forgiveness. For the current sample, the Cronbach’s *α*’s for the DFS were 0.63 and 0.82 for husbands and wives at T1 respectively.

#### Emotional Forgiveness

The 8-item self-report Emotional Forgiveness Scale (EFS; Worthington et al., 2007) was used to measure the degree to which one had experienced emotional forgiveness and peace for a specific offense. Participants indicated their agreement with each item on a 5-point rating scale, from 1 (strongly disagree) to 5 (strongly agree). The EFS has two 4-item subscales, one indicating the reduction of negative emotions toward an offender (e.g., “I no longer feel upset when I think of him or her”), and one indicating the presence of positive emotions toward an offender (e.g., “I feel sympathy toward him or her”). Mean scores were calculated and used in analyses. Higher scores indicated higher levels of emotional forgiveness. For the current sample, the Cronbach’s *α*’s for the EFS were 0.76 and 0.79 for husbands and wives at T1 separately. Finally, Items from DFS and EFS have been used in other studies focusing on forgiveness within romantic relationships (e.g., Chi et al., 2011), and have shown good internal consistency.

### Data Analysis

Hypotheses were tested with structural equation modeling in Mplus Version 7.0 (Muthén and Muthén, 2010). The adequacy of models was evaluated using the following indices (Kline, 2011): the chi-square statistic, the comparative fit index (CFI), the root mean square error of approximation (RMSEA), and the standardized root-mean- square residual (SRMR). Models with non-significant chi-square values, CFI > 0.95, RMSEA < 0.05, and SRMR < 0.05 were considered to have an excellent fit. Last, missing values were addressed by using the full information maximum likelihood (FIML) estimation method (Schafer and Graham, 2002).

When testing the hypothesized mediational model, we followed the actor–partner interdependence mediation model (APIMeM; Ledermann et al., 2011). Specifically, we examined the associations between forgiveness and both concurrent and longitudinal levels in marital stability. A concurrent actor–partner interdependence mediation model was tested in which spouses’ decisional and emotional forgiveness at T1 was linked to their own and partners’ marital stability at T1 through their own and partners’ marital quality at T1. A longitudinal actor–partner interdependence mediation model was tested in which spouses’ decisional and emotional forgiveness at T1 influences their own and partners’ marital stability at T3 through their own and partners’ marital quality at T2. To account for the autoregressive, stability effects, the baseline levels of spouses’ marital quality and marital stability at Time 1 were included in the model as exogenous variables predicting their own marital quality at Time 2 and marital stability at Time 3, respectively. To account for the possible dependency in couple dyadic data, correlated residuals were also specified in the model linking husbands’ decisional/emotional forgiveness to wives’ decisional/emotional forgiveness, husbands’ marital quality to wives’ marital quality, and husbands’ marital stability to wives’ marital stability. There was a significant bivariate correlation found between marital duration and marital quality. Thus, we controlled for marital duration in all analyses. Lastly, indirect effects in each model were estimated using a 5,000-sample, bias-corrected bootstrapping procedure (Hayes, 2013) with a 95% confidence interval.

## Results

### Preliminary and Descriptive Statistics

Descriptive statistics for all study variables and the reliabilities for each measure are shown in **Table 1**. We found that almost all key variables were positively correlated, with the only exception of the husbands’ and wives’ decisional forgiveness and their partner’s marital quality and stability, which were not significantly correlated.

**Table 1:** Descriptive statistics and correlations for the study variables.

	1	2	3	4	5	6	7	8	9	10	11	12	13
(1) H DF T1	1												
(2) W DF T1	0.02	1											
(3) H EF T1	0.48^∗∗^	0.03	1										
(4) W EF T1	0.07	0.53^∗∗^	0.22^∗∗^	1									
(5) H MQ T1	0.31^∗∗^	0.09	0.45^∗∗∗^	0.29^∗∗^	1								
(6) W MQ T1	0.10	0.22^∗∗^	0.27^∗∗^	0.51^∗∗^	0.43^∗∗^	1							
(7) H MS T1	0.37^∗∗^	0.14^∗^	0.43^∗∗∗^	0.33^∗∗^	0.54^∗∗∗^	0.44^∗∗∗^	1						
(8) W MS T1	0.05	0.30^∗∗^	0.22^∗∗^	0.43^∗∗∗^	0.35^∗∗^	0.60^∗∗∗^	0.53^∗∗^	1					
(9) H MQ T2	0.08	0.05	0.27^∗∗^	0.22^∗∗^	0.32^∗∗^	0.18^∗∗^	0.32^∗∗^	0.16^∗∗^	1				
(10) W MQ T2	0.02	0.17^∗^	0.14^∗^	0.36^∗∗^	0.16^∗∗^	0.32^∗∗^	0.27^∗∗^	0.48^∗∗∗^	0.52^∗∗∗^	1			
(11) H MS T3	0.26^∗∗^	0.02	0.40^∗∗∗^	0.23^∗∗^	0.41^∗∗∗^	0.27^∗∗^	0.62^∗∗^	0.32^∗∗^	0.55^∗∗∗^	0.50^∗∗∗^	1		
(12) W MS T3	0.15^∗^	0.08	0.24^∗∗^	0.28^∗∗^	0.19^∗∗^	0.24^∗∗^	0.27^∗∗^	0.40^∗∗^	0.32^∗∗^	0.69^∗∗∗^	0.61^∗∗∗^	1	
(13) MD	-0.05	-0.03	-0.08	-0.05	-0.10	-0.10	-0.24^∗∗^	-0.16^∗∗^	-0.24^∗∗^	-0.21^∗∗^	-0.24^∗∗^	-0.25^∗∗^	
*M*	4.31	4.13	4.14	3.94	6.73	6.62	3.81	3.74	6.53	6.32	3.72	3.62	13.63
*SD*	0.49	0.53	0.59	0.56	0.96	1.10	0.34	0.41	1.40	1.29	0.46	0.53	9.67

*H, husbands; W, wives; DF, decisional forgiveness; EF, emotional forgiveness; MQ, marital quality; MS, marital stability; MD, marital duration; T, time. *^∗^p <* 0.05. *^∗∗^p <* 0.01. *^∗∗∗^p* < 0.001 (two-tailed)*.

Paired *t*-tests were conducted to reveal the differences between wives and husbands on key variables. Husbands reported greater decisional forgiveness (*t*_267_ = 3.45, *p* < 0.001) and emotional forgiveness (*t*_267_ = 4.65, *p* < 0.001) at T1, and greater marital stability at T1 (*t*_267_ = 2.99, *p* < 0.01) and T3 (*t*_200_ = 2.67, *p* < 0.01) than wives. Spouses did not differ from each other in marital quality at T1 or T2.

### Concurrent Models

**FIGURE 1:**
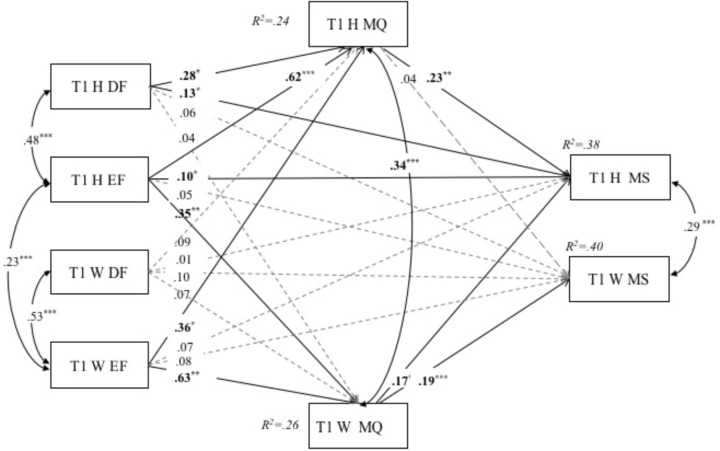
Actor–partner mediation model for Tl decisional forgiveness, emotional forgiveness, marital quality, and marital stability. Figure values are unstandardized regression coefficients. H, husbands; W, wives; DF, decisional forgiveness; EF, emotional forgiveness; MQ, marital quality; MS, marital stability; T, time. *^∗^p <* 0.05. ^∗^*p* < 0.01. ^∗∗^*^∗^p* < 0.001.

The APIMeM is presented in **Figure 1**. The full model provided a good fit to the data, χ^2^(0) = 0, *p* = 0, RMSEA = 0, CFI = 1, SRMR = 0. As evident in **Figure 1**, there were significant direct effects of decisional forgiveness (*β* = 0.18, *p* < 0.01) on marital stability, and direct effects of emotional forgiveness (*β* = 0.15, *p* < 0.05) on marital stability for husbands.

Results of the all indirect effects in the analyzed model are presented in **Table 2**. As shown in the **Table 2**, three significant indirect actor effects were identified: (a) husbands’ decisional forgiveness → husbands’ marital quality → husbands’ marital stability (*β* = 0.03, 95% CI [0.01,0.06]); (b) husbands’ emotional forgiveness → husbands’ marital quality → husbands’ marital stability (*β* = 0.05, 95% CI [0.01,0.12],); (c) wives’ emotional forgiveness → wives’ marital quality → wives’ marital stability (*β* = 0.17, 95% CI [0.10,0.29]). **Table 2** also revealed four statistically significant indirect partner effects: (a) husbands’ emotional forgiveness → wives’ marital quality → husbands’ marital stability (*β* = 0.02, 95% CI [0.003,0.05]); (b) husbands’ emotional forgiveness → wives’ marital quality → wives’ marital stability (*β* = 0.07, 95% CI [0.02,0.14]); (c) wives’ emotional forgiveness → wives’ marital quality → husbands’ marital stability (*β* = 0.05, 95% CI [0.01,0.10]); and (d) wives’ emotional forgiveness → husbands’ marital quality → husbands’ marital stability (*β* = 0.03, 95% CI [0.01,0.08]).

### Longitudinal Models

**FIGURE 2:**
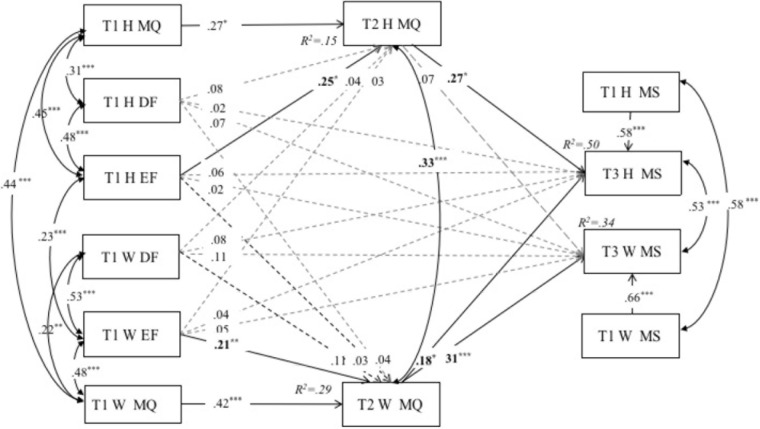
Actor–partner mediation model for Tl decisional forgiveness and emotional forgiveness, T2 marital quality and T3 marital stability. Figure values are unstandardized regression coefficients. H, husbands; W, wives; DF, decisional forgiveness; EF, emotional forgiveness; MQ, marital quality; MS, marital stability; T, time, *^∗^p* < 0.05. *^∗∗^p <* 0.01. *^∗∗∗^p <* 0.001.

Based on the concurrent model, we kept all paths shown in **Figure 1** but substituted T2 marital quality for T1 marital quality as a mediator, and controlled for the associations between spouses’ T1 and T2 marital quality. We also substituted T3 marital stability for T1 marital stability as an outcome variable, and controlled for the associations between spouses’ T1 and T3 marital stability. The APIMeM, with direct effects between the independent and outcome variables, is presented in **Figure 2**. The full model provided a good fit to the data, χ^2^ (16) = 29.41, *p* < 0.05, RMSEA = 0.05 (90% CI = 0.02,0.09), CFI = 0.97, SRMR = 0.04.

Results of the associated indirect effects in the analyzed model are presented in **Table 3**. As shown in the **Table 3**, two significant indirect actor effects were identified: (a) T1 husbands’ emotional forgiveness → T2 husbands’ martial quality → T3 husbands’ martial stability (*β* = 0.12, 95% CI [0.01,0.35]); (b) T1 wives’ emotional forgiveness → T2 wives’ martial quality → T3 wives’ martial stability (*β* = 0.31, 95% CI [0.11,0.65]). **Table 3** also reveals a statistically significant indirect partner effects from wives’ emotional forgiveness → wives’ marital quality → husbands’ marital stability (*β* = 0.14, 95% CI [0.03,0.37]).

**Table 2:** Indirect effects from the Actor–Partner Mediator Model with T1 decisional forgiveness and emotional forgiveness as independent variable, marital quality as mediator, and marital stability as dependent variable.

Effect		IE	95% CI
**D_H1_ → M_H1_ → Y_H1_**	**= A → A**	**0.03**	**[0.01,0.06]**
**E_H1_ → M_H1_ → Y_H1_**	**= A → A**	**0.05**	**[0.01,0.12]**
D_W1_ **→** M_W1_ **→** Y_W1_	= A **→** A	0.01	[-0.07,0.04]
**E_W1_ → M_W1_ → Y_W1_**	**= A → A**	**0.17**	**[0.10,0.29]**
D_H1_ **→** M_W1_ **→** Y_H1_	= P **→**P	0.002	[-0.02,0.01]
**E_H1_ → M_W1_ → Y_H1_**	**= P → P**	**0.02**	**[0.003,0.05]**
D_W1_ **→** M_H1_ **→** Y_W1_	= P **→** P	0.004	[-0.03,0.003]
E_W1_ **→** M_H1_ **→** Y_W1_	= P **→** P	0.02	[-0.03,0.006]
D_H1_ **→** M_H1_ **→** Y_W1_	= A**→** P	0.01	[-0.003,0.05]
E_H1_ **→** M_H1_ **→** Y_W1_	= A**→** P	0.03	[-0.01,0.09]
D_W1_ **→** M_W1_ **→** Y_H1_	= A**→** P	0.01	[-0.02,0.09]
**E_W1_ → M_W1_ → Y_H1_**	**= A→ P**	**0.05**	**[0.01,0.10]**
D_H1_ **→** M_W1_ **→** Y_W1_	= P **→**A	0.01	[-0.06,0.04]
**E_H1_ → M_W1_ → Y_W1_**	**= P →A**	**0.07**	**[0.02,0.14]**
D_W1_ **→** M_H1_ **→** Y_H1_	= P **→** A	0.01	[-0.03,0.09]
**E_W1_ → M_H1_ → Y_H1_**	**= P →A**	**0.03**	**[0.01,0.08]**

*Table values are unstandardized coefficients. IE, indirect effect; CI, confidence interval; DF, Decisional Forgiveness; EF, Emotional Forgiveness; H, husband; W, wife; A, actor effect; P, partner effect. Bold values signifies significant pathways with parameter estimates with p < 0.05*.

## Discussion

This study was among the first to apply a dyadic approach and a longitudinal design to test the predictions that decisional forgiveness and emotional forgiveness would be positively associated with marital stability, and that the relationships would be explained by marital quality within the context of the early years of Chinese marriage. This study not only extends previous research by examining the associations among forgiveness, marital quality, and marital stability from a broader theoretical framework of the VSA model, but also identified that emotional forgiveness, rather than decisional forgiveness, is one of the most important contributing factors of long-term marital stability.

As predicted, decisional forgiveness and emotional forgiveness were positively associated with the concurrent level of marital stability for husbands and wives separately. This finding supports prior research indicating that forgiveness is featured as a successful relational repair process following transgressions (Rusbult et al., 2005; Entezar et al., 2011; Merolla et al., 2013) linked with relationship stability (Fincham et al., 2007; David and Stafford, 2015; Kato, 2016). When the impact of marital quality was accounted for, the direct effects of decisional forgiveness on the concurrent level of marital stability was only significant for husbands. The lack of an effect of decisional forgiveness on the concurrent level of marital stability for wives could reflect differences in how men and women manage forgiveness experiences and responses to transgression in their romantic relationships. Prior research documents that women value affection and love (Hook et al., 2003). It may thus be the case that since women are socialized to perform more emotional work, such as expressing more hurt, disappointment, and sadness, in a marriage, when engaged in attempts to resolve marital conflict (Brody, 1993; Canary and Wahba, 2006), women may thus focus less upon decisional forgiveness than men. In other words, women’s forgiveness may be more triggered by positive feelings, rather than behavioral choices toward the transgressors.

Our results suggest that decisional forgiveness does not significantly contribute to marital stability through marital quality over time for husbands and wives. Such effects may be primarily accounted for by two reasons. First, as some scholars contended (Enright, 1996; Hook et al., 2009), decisional forgiveness in the form of unforgiveness reduction alone is insufficient to maintain long-term relationship stability. Second, given that spouses in the present study are from a collectivistic culture, the social pressure may motivate them to make decisions to forgive in order to maintain group harmony (Hook et al., 2009), even when they may still be emotionally unforgiving (i.e., holding a grudge against the transgressor). This phenomenon is regarded as “hollow forgiveness” (Baumeister et al., 1998).

**Table 3:** Indirect effects from the Actor–Partner Mediator Model with T1 emotional forgiveness as independent variable, T2 marital quality as mediator, and T3 marital stability as dependent variable.

Effect		IE	95%CI
D_H1_ **→** M_H2_ **→** Y_3_	= A **→** A	0.04	[-0.16,0.07]
**E_H1_ → M_H2_→ Y_H3_**	**= A → A**	**0.12**	**[0.01,0.35]**
D_W1_ **→** M_W1_ **→** Y_W1_	= A **→** A	0.01	[-0.19,0.18]
**E_W1_ → M_W2_→ Y_W3_**	**= A → A**	**0.31**	**[0.11,0.65]**
D_H1_ **→** M_W1_ **→** Y_H1_	= P **→**P	0.02	[-0.14,0.08]
E_H1_ **→** M_W2_ **→** Y_H3_	= P **→** P	0.02	[-0.08,0.14]
D_W1_ **→** M_H1_ **→** Y_W1_	= P **→** P	0.01	[-0.11,0.03]
E_W1_ **→** M_H1_ **→** Y_W1_	= P **→** P	0.05	[-0.02,0.24]
D_H1_ **→** M_H1_ **→** Y_W1_	= A**→** P	0.02	[-0.19,0.03]
E_H1_ **→** M_H1_ **→** Y_W1_	= A**→** P	0.06	[-0.02,0.29]
D_W1_ **→** M_W1_ **→** Y_H1_	= A**→** P	0.01	[-0.10,0.08]
**E_W1_ → M_W2_ → Y_H3_**	**= A→ P**	**0.14**	**[0.03,0.37]**
D_H1_ **→** M_W1_ **→** Y_W1_	= P **→**A	0.04	[-0.28,0.19]
E_H1_ **→** M_W2_ **→** Y_W3_	= P **→**A	0.04	[-0.20,0.27]
D_W1_ **→** M_H2_ **→** Y_H3_	= P **→**A	0.02	[-0.11,0.08]
E_W1_ **→** M_H2_ **→** Y_H3_	= P **→**A	0.08	[-0.01,0.25]

*Table values are unstandardized coefficients. IE, indirect effect; CI, confidence interval; EF, Emotional Forgiveness; H, husband; W, wife; A, actor effect; P, partner effect. Bold values signifies significant pathways with parameter estimates with p < 0.05.*

We hypothesized that emotional forgiveness, rather than decisional forgiveness would be positively related to the longitudinal levels of marital stability through marital quality. Our results provided good evidence for this hypothesis. In the concurrent model, we found that emotional forgiveness was indirectly associated with marital stability through marital quality for both husbands and wives. These findings were replicated in the longitudinal model, and then allow stronger inferences to be drawn about the direction of the indirect effects of emotional forgiveness on subsequent marital stability via marital quality. As noted already, emotional forgiveness involves changing emotion, cognition, and motivation to eliminate unforgiveness (Worthington, 2005), and thereby serve the adaptive function to mobilize energy and give direction to preserve a long-lasting relationship. Moreover, in ongoing relationships, such as marriage, injuries and transgressions are inevitable and may negatively influence marital stability. As a result, Fincham and colleagues indicated that reduction in negative motivation toward the transgressor is important in order to promote relationship repair following a transgression; however, emotional forgiveness plays a salient role in maintaining long-term stable marriages when the transgressor is one’s intimate partner (Fincham et al., 2007), since it cultivates a positive affect by replacing negative emotions that eventually build an aggregate positive experience toward one’s partner (Worthington, 2005). As a result, it may be an important first step to decide to forgive a transgressor, but in order to truly forgive, one has to also emphasize emotional forgiveness and feel positive affect with the transgressor (Lichtenfeld et al., 2015).

Interestingly, it appears that the partner results were mixed. For wives, their marital quality mediated the association between their emotional forgiveness and husbands’ marital stability. The findings were robust when examined concurrently and longitudinally. That was not the case for husbands in the longitudinal model, for whom there were no significant partner effects. We believe that the different results for husbands and wives reflects the widespread view that women, who are thought to more attuned to relationships than men, place a greater salience and importance on relationships, and often serve as the barometers for the functioning of the relationship (Fincham and Linfield, 1997). Thus, it is not surprising that the wives’ emotional forgiveness was indirectly associated with the husbands’ marital stability through wives’ marital quality. In addition, this finding may reflect the situation that Chinese women’s status has been changing in the last decades. Historically, women have been socialized to facilitate interpersonal relationships (Wink and Helson, 1993). However, Chinese women’s status has been improved by the revision of marriage laws (e.g., “1950 Marriage Law”), which acknowledged individual freedom and promoted marital equality rights (Davis, 2014), and the vast majority of Chinese young women were born after the aforementioned introduction of “1979 One-Child Policy,” which may contribute to their focus on self-interests and the expression of emotion. Chinese women who have recently tapped into the feminist consciousness due to the introduction of Western marital culture, especially those highly educated (as was the case in the present study), may emphasize equality and personal happiness when entering marriage (Xu et al., 2007). This scenario may explain why women’s emotional forgiveness related to perceptions of marital quality, which in turn, resulted in their own, as well as their spouse’s marital stability. In addition, as the attrition analyses indicated, the retained husbands reported higher levels of marital quality and marital stability than did the attrited husbands. That is, given that husbands who were not that satisfied in marriage were more likely to withdraw from the later participation, couples retained at the later waves may represent a group with lower levels of marital risks, especially for husbands. From a methodological perspective, this may limit the variance of the changes in husbands’ marital quality and marital stability across waves, and also may be partly responsible for the absence of significant indirect partner effects on the husband side.

In general, the results presented here extend several aspects of previous research and provide some insights. This was the first study to examine longitudinal associations between couple forgiveness and marital stability, as well as the effect of marital quality on the relationship between forgiveness and marital stability in the APIMeM. We found that emotional forgiveness (as a cognitive and emotional component involved in couple adaptive processes) was associated with marital stability indirectly through marital quality. These findings provide empirical evidence supporting one of the core propositions of the VSA model that “spouses’ ability to adapt to the challenges they encounter may be associated with the subsequent marital stability through marital quality” (Karney and Bradbury, 1995; i.e., adaptive processes → marital quality → marital stability). However, given that the VSA model was developed based on research conducted with samples of Western couples from individualistic cultures, more efforts are needed to test the applicability of the other pathways in the VSA model to Chinese couple.

Notwithstanding the important advances represented by the current findings, several limitations of the research need to be acknowledged. First, as this study used correlational, self-report data, it is critical to develop ways of investigating the mechanisms identified using experimental methods in future research. Second, this study’s sample was a group of newlyweds in China who had higher levels of income and education than the broader population. Thus, the extent to which the findings can be generalized beyond our sample is unclear, and research is needed to investigate forgiveness in couples in different marital stages and socioeconomic status (SES) levels.

Third, those husbands who were reported lower levels of marital quality and marital stability tended to drop out of the study, and thus our sample underrepresented husbands who were initially located in the lower end of the marital quality and marital stability distributions and our estimated coefficients may have represented a lower bound in the strength of the relationship on the husband side. Fourth, it is also worth noting that we focused on the offense-specific assessments of forgiveness (i.e., situational forgiveness) in this study. Future research will benefit from conducting studies with longitudinal designs to examine whether the findings of the present study are generalizable to dispositional forgiveness (e.g., tendency to forgive). Lastly, we considered self-perceived forgiveness between the two spouses, instead of their partners’ actual forgiveness, which may better account for marital stability than self-perceived forgiveness from a dyadic perspective. Future studies should address this issue, testing whether the perceptions of a partner’s forgiveness predict marital stability, and whether marital quality plays a mediated role in this association.

## Ethics Statement

This studywas carried out in accordance with the recommendations of Ethics Review Committee at the School of Psychology, BNU with written informed consent from all subjects. All subjects gave written informed consent in accordance with the Declaration of Helsinki. The protocol was approved by the Ethics Review Committee at the School of Psychology, BNU.

## Author Contributions

QH, MZ, and XF designed this study and drafted the manuscript. QH, MZ, WT, JL, XL, and XJ performed the research. QH, MZ, and XF analyzed the data. All the authors approved the final version of the manuscript for submission.

## Conflict of Interest Statement

The authors declare that the research was conducted in the absence of any commercial or financial relationships that could be construed as a potential conflict of interest.
